# Supplementary material for: Effective Prediction of Prostate Cancer Recurrence through the IQGAP1 Network
Source: Cancers (Basel). 2021 Jan 23;13(3):430. doi: 10.3390/cancers13030430 (PMC7865788; doi:10.3390/cancers13030430)
Supplement: Supplementary file 1 [file cancers-13-00430-s001.zip › Fig S1.pdf]

Figure S1

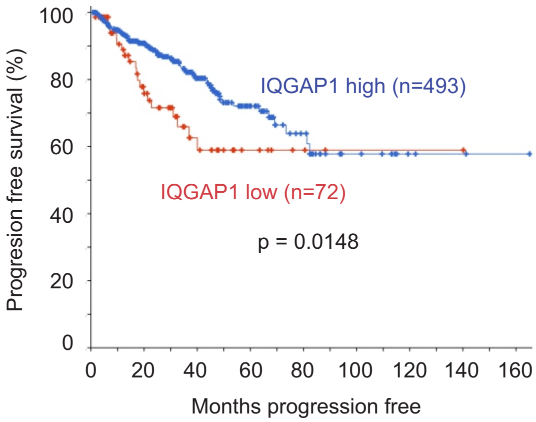

Figure S1. Downregulation of IQGAP1 is associated with PC recurrence. The TCGA PanCancer Atlas PC dataset was divided into a high and low relapse risk group following prostatectomy using the cutoff point of -1SD (standard deviation). Kaplan Meier survival curve and lograk test were performed using tools provided by cBioPortal.
