# Supplementary material for: Effective Prediction of Prostate Cancer Recurrence through the IQGAP1 Network
Source: Cancers (Basel). 2021 Jan 23;13(3):430. doi: 10.3390/cancers13030430 (PMC7865788; doi:10.3390/cancers13030430)
Supplement: Supplementary file 1 [file cancers-13-00430-s001.zip › Fig S2.pdf]

Figure S2

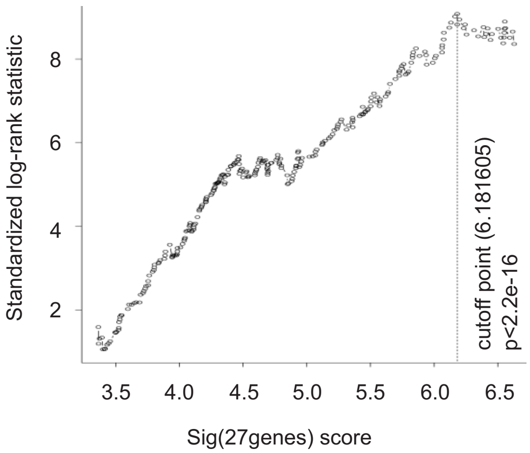

Figure S2. Cutoff point estimation for Sig27gene in stratifying PC recurrence. The cutoff point of Sig27gene scores was determined using Maximally Selected Rank Statistics (the *Maxstat* package) in R. the cutoff point and its associated p values are indicated.
