# Supplementary material for: Effective Prediction of Prostate Cancer Recurrence through the IQGAP1 Network
Source: Cancers (Basel). 2021 Jan 23;13(3):430. doi: 10.3390/cancers13030430 (PMC7865788; doi:10.3390/cancers13030430)
Supplement: Supplementary file 1 [file cancers-13-00430-s001.zip › Fig S3.pdf]

Figure S3

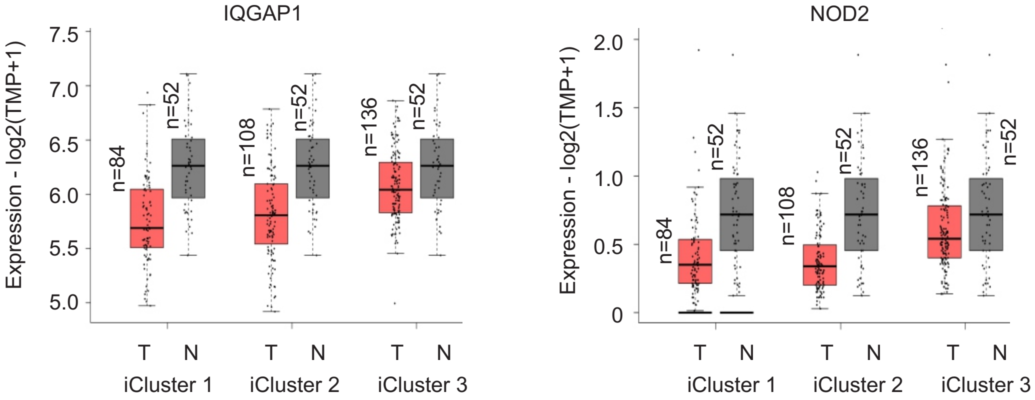

Figure S3. Expression of IQGAP1 and NOD2 in PCs vs matched normal prostate tissues. The analyses were performed using the GEPIA2 program. T: tumor; N: normal tissues; TPM: transcripts per million. Statistical analyses (one-way ANOVA) were performed by GEPIA2.
