# Supplementary material for: Effective Prediction of Prostate Cancer Recurrence through the IQGAP1 Network
Source: Cancers (Basel). 2021 Jan 23;13(3):430. doi: 10.3390/cancers13030430 (PMC7865788; doi:10.3390/cancers13030430)
Supplement: Supplementary file 1 [file cancers-13-00430-s001.zip › Fig S4.pdf]

Figure S4

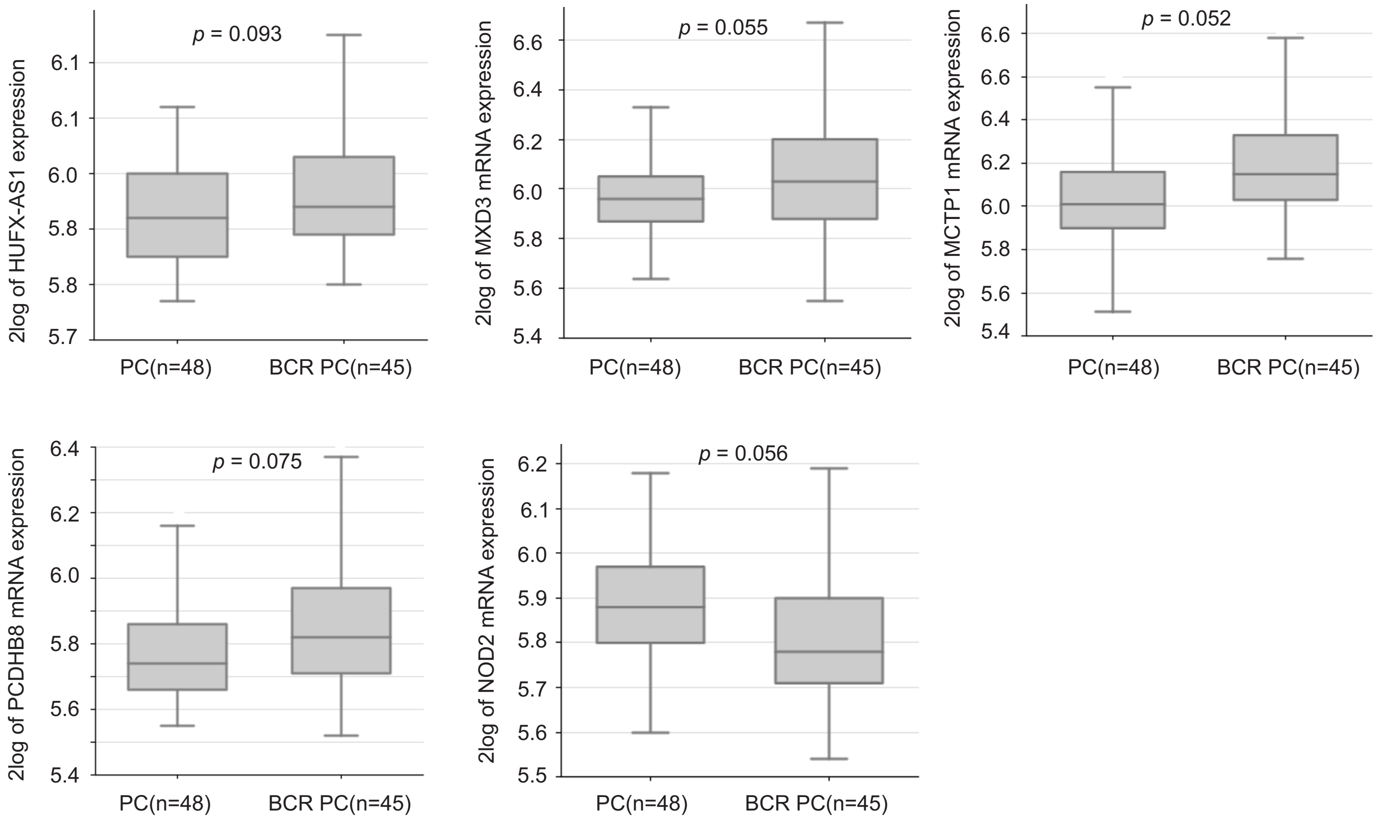

Figure S4. Expression of the indicated Sig27gene component genes in primary PCs with BCR development (BCR PC) and without the progression (PC). Analyses were performed using the Dunning dataset in R2: Genomics Analysis and Visualization Platform. Gene expression in the dataset was determined using microarray. The expression was presented as log2-transformed data. Statistical analyses were performed by the R2 Platform using one-way ANOVA.
