# Supplementary material for: Effective Prediction of Prostate Cancer Recurrence through the IQGAP1 Network
Source: Cancers (Basel). 2021 Jan 23;13(3):430. doi: 10.3390/cancers13030430 (PMC7865788; doi:10.3390/cancers13030430)
Supplement: Supplementary file 1 [file cancers-13-00430-s001.zip › Fig S5.pdf]

Figure S5

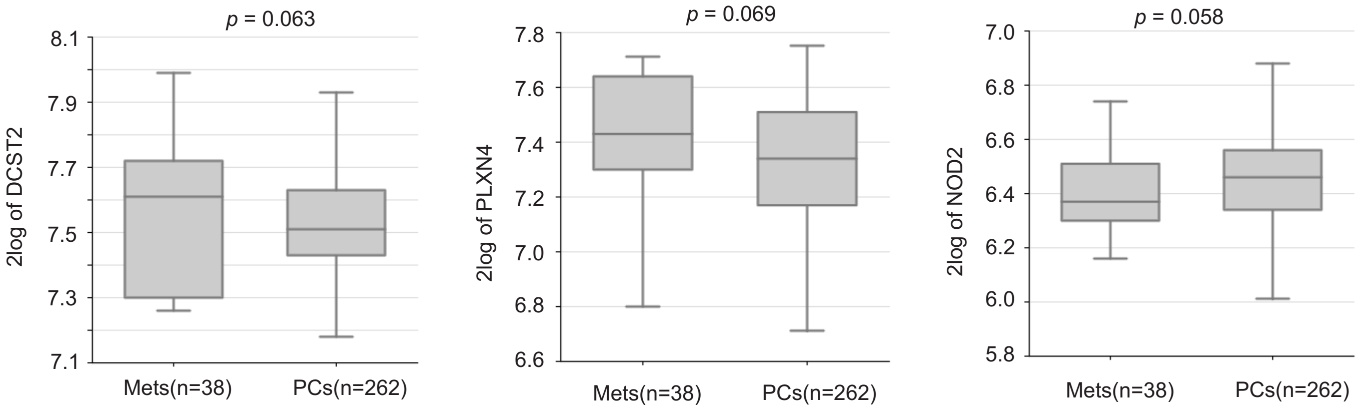


**Figure S5.** Expression of the indicated Sig27gene component genes in metastatic PCs (Mets) compared to primary PCs (PCs) using the Sawyers dataset in R2: Genomics Analysis and Visualization Platform. Gene expression in the dataset was determined using microarray. The expression of the indicated genes was presented as log2-transformed data. Statistical analyses were performed by the R2 Platform using one-way ANOVA.
