# Supplementary material for: Effective Prediction of Prostate Cancer Recurrence through the IQGAP1 Network
Source: Cancers (Basel). 2021 Jan 23;13(3):430. doi: 10.3390/cancers13030430 (PMC7865788; doi:10.3390/cancers13030430)
Supplement: Supplementary file 1 [file cancers-13-00430-s001.zip › Table S1.docx]

**Table S1**. Clinical prostate cancer tissues used in IHC staining for IQGAP1

| **Patient** | **Age ^a^** | **Gleason Score** | **Average H-Score ^b^** |
| --- | --- | --- | --- |
| 1 | 68 | 4+4 | 88.8411 |
| 2 | 76 | 5+5 | 22.5717 |
| 3 ^c^ | 86 | 5+5 | 11.0301 |
| 4 | 75 | 4+5 | 68.914 |
| 5 | 81 | 5+5 | 44.0166 |
| 6 | 49 | 4+5 | 27.6064 |
| 7 | 66 | 4+5 | 9.2196 |
| 8 | 64 | 4+5 | 1.4702 |
| 9 | 71 | 4+5 | 3.294 |
| 10 | 69 | 4+5 | 46.1535 |
| 11 | 70 | 4+5 | 41.1938 |
| 12 | 91 | 4+5 | 6.7122 |
| 13 | 83 | 4+5 | 25.8473 |
| 14 | 61 | 5+4 | 38.9899 |
| 15 | 62 | 3+3 | 99.8332 |
| 16 | 72 | 3+3 | 141.4827 |
| 17 | 78 | 3+3 | 59.7504 |
| 18 | 70 | 3+3 | 35.5889 |
| 19 | 56 | 3+3 | 37.2519 |
| 20 ^c^ | 69 | 2+3 | 88.8411 |
| 21 | 55 | 3+3 | 36.8144 |
| 22 | 78 | 3+3 | 69.7004 |
| 23 | 75 | 3+3 | 54.5919 |
| 24 | 73 | 3+3 | 21.4627 |
| 25 | 78 | 3+4 | 59.2938 |
| 26 | 69 | 4+3 | 82.514 |
| 27 | 78 | 4+4 | 68.914 |

a: age at diagnosis; b: quantification of IQGAP1 staining; c: tissues obtained from TURP (transurethral resection of the prostate)
